# Impact of pen move events on rumination time, milk yield, and water intake in dairy cows

**DOI:** 10.3168/jdsc.2025-0904

**Published:** 2026-02-06

**Authors:** Rajesh Neupane, Natalie Fagundo, Sushil Paudyal

**Affiliations:** Department of Animal Science, Texas A&M University, College Station, TX 77843

## Abstract

Cow regrouping associated with pen move events (PME) within a dairy farm disrupts the social dynamics of cows in the herd and affects cow behavior and performance. The objective of this study was to evaluate the effect of PME and cow move group size on rumination time (RT; min/d), milk yield (MY; kg/d), and water intake (WI; L/d) of dairy cows up to 5 d postmove. A retrospective study was conducted on an organic dairy farm in central Texas, which consisted of 5,420 independent cow move events collected from 1,130 crossbred dairy cows. Cow-related variables were retrieved from herd management software, daily RT and WI were retrieved from sensor boluses, and daily MY was retrieved from milk meters on the farm. Statistical analyses were conducted in R using repeated measures in a mixed-effects model controlled for stage of lactation, temperature-humidity index, and parity, with cow as a random effect. We identified a 3-way interaction effect between parity, stage of lactation, and day since the pen move on RT. During early lactation, the reduction in RT was more pronounced in primiparous cows on d 2 to d 5 post-PME compared with d 1. In multiparous cows, during the dry period, RT was significantly reduced compared with d 1. Similarly, MY decreased during early lactation in multiparous cows and during late lactation in primiparous cows. A parity by stage of lactation by day 3-way interaction was observed in WI, with decreased WI in primiparous cows during early lactation after PME. In contrast, WI increased in multiparous cows in the dry period and mid lactation. Furthermore, WI, RT, and MY were also affected by the size of the cow move groups (single, small, or large) and varied across different parities; primiparous cows moved in larger groups showed a 10% drop in RT compared with a 6% drop in multiparous cows moved in large groups. Compared with primiparous cows, multiparous cows experienced a more pronounced decline in MY when moved in small or large groups, along with a significant reduction in WI in the large-group moves. We demonstrate that daily RT, MY, and WI are affected by PME, with the effect dependent on the parity of the cows and the size of the cow move group.

## Highlights

• Effects of pen move events vary by parity, lactation stage, and group size.
• Primiparous cows showed pronounced declines in early lactation for rumination time.
• Primiparous cows showed a decrease in MY and WI following large group moves.
• Multiparous cows experienced notable decreases in rumination during the dry period.
• Multiparous cows experienced a decrease in milk yield and water intake in large group moves.

## Graphical Abstract

**Figure:**
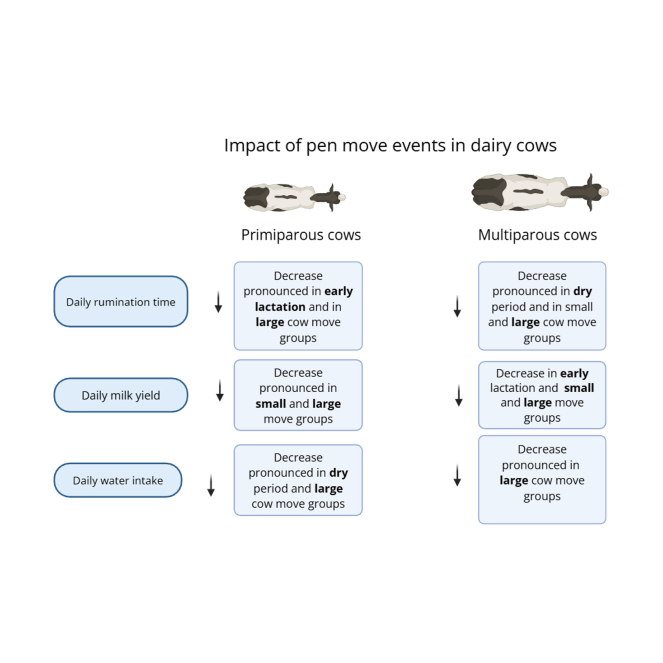
**Summary:** This study evaluated the effects of pen move events (PME) on dairy cow rumination time (RT), milk yield (MY), and water intake (WI) up to 5 days after regrouping on a commercial organic dairy farm. Using sensor boluses and herd management data, the study analyzed 5,420 PME involving 1,130 crossbred cows. Results revealed that PME significantly reduced RT, MY, and WI, with pronounced effects varying by parity (primiparous vs. multiparous), lactation stage, and move group size. Early-lactation primiparous cows experienced notable declines in RT and WI, especially following large-group moves, whereas multiparous cows showed the greatest reductions during the dry period and after large-group PME. Both RT and MY were significantly reduced for several days postmove, indicating social stress and behavioral disruption.

Pen move events (**PME**) and associated cow regrouping are common in dairy farming to manage herd composition and stocking density in a pen. This common practice in dairy farms helps to align cow groups by parity, lactation stage, or production level to meet the management needs for group-specific feed rations and special handling requirements (Nordlund et al., 2006). A USDA National Animal Health Monitoring System (USDA APHIS, 2014) study of US dairy farms reported that 63% of large dairy farms feed different rations according to lactation number, stage of lactation, or production level, which consequently results in cows moving from one group to another during their lactation. Occurring up to 4 to 5 times during a lactation cycle, more frequent pen moves can stress cows by altering social hierarchies built up within each pen, potentially leading to increased competition and stress, which can affect cow behavior and performance (Grant and Albright, 2001; Nordlund et al., 2006; Foris et al., 2021). Previous studies have shown that PME often reduce rumination time (**RT**) and milk yield (**MY**), particularly affecting younger, primiparous cows more than older, multiparous ones (Grant and Albright, 2001; Marumo et al., 2024). Rumination is commonly used as an indicator of rumen health in dairy cows because it supports nutrient absorption and overall health. Previous studies that reported reduction in RT following PME (Schirmann et al., 2011; Marumo et al., 2024) have suggested social stress and competition for resources as a cause of short-term disturbances in rumination function. However, the response of cows on days immediately following PME has not been widely documented. The stress related to PME has been shown to negatively affect MY (Scheurwater et al., 2021; Marumo et al., 2024). Although these observations are generally associated with resource constraints and associated competitions, studies evaluating water intake (**WI**) during the move events are scarce. Furthermore, the effects of the move group size during PME have not been extensively studied. Therefore, the objective of this study was to quantify the effect of PME and move group size on RT, MY, and WI of dairy cows up to 5 d after PME.

This retrospective observational study was conducted on a USDA-certified organic dairy farm located in central Texas, milking ∼2,500 crossbred dairy cows (Holstein × Jersey crossbred). The cows were housed in 8 pens, each accommodating ∼300 cows. The farm aimed to maintain milking pens at 100% stocking density, while the fresh cow and hospital pens were kept at 80% stocking density in both feeding and lying areas. Cow-level PME data were collected from January 2022 to December 2024, encompassing 5,420 independent PME from 1,130 lactating cows. A PME was defined as the relocation of an individual cow to a different pen within the farm, typically for management purposes such as grouping by lactation stage, parity, health status, reproduction status, or production level. The exclusion criteria included cows moving to hospital pens for treatment and hoof trimmings, as well as cow move events taking place during the annual grazing period (May–August). Cow information related to PME, including parity (primiparous, n = 396; multiparous n = 845), stage of lactation (early, n = 912: 1–100 DIM; mid, n = 197: 101–200 DIM; late, n = 153: >200 DIM; dry, n = 403), and PME dates, were retrieved from herd management software (DairyComp 305, Valley Agricultural Software, Tulare, CA). Daily RT and WI were measured using SmaXtec sensor boluses (SmaXtec Animal Care GmbH, Graz, Austria), which were implanted in each cow to continuously monitor rumen activity and water consumption. The SmaXtec system provided RT as total minutes ruminating per day and WI as liters consumed per day. Cows were milked 2 times a day, and MY (kg/d) was recorded using Afimilk milk meters (Afimilk Ltd., Kibbutz Afikim, Israel) installed in the milking parlor, which measured milk production per cow per milking session and aggregated the values to daily totals. Environmental data, specifically daily temperature and humidity, were collected from a nearby weather station using a Python API to calculate the temperature-humidity index (**THI**) using the equation: THI = (1.8 × T + 32) − [(0.55 − 0.0055 × RH) × (1.8 × T − 26)] (Kelly et al., 1971), where T is the ambient temperature (°C) and RH is the relative humidity (%). Daily THI values were used to account for environmental effects on cow performance. Data were extracted for a 7-d timeframe window around each PME, including the day before the move (d −1), the day of the move (d 0), and 5 d postmove (d 1–5). Only PME with complete data for RT, MY, WI, and cow-related variables were included in the analysis. Furthermore, a moved cow group size variable was created, which identified how many cows were moved together from the same origin pen to the same destination pen on the day of PME. The effect of PME group size was evaluated by categorizing moves as single-cow PME (n = 2,531), small groups (2–7 cows; n = 1,623), and large groups (>7; n = 1,263) moving between destination and source pen on a given day based on the farm protocol and management relevance.

Data from DairyComp 305, SmaXtec, and Afimilk systems were merged using unique cow identification numbers and timestamps. Data cleaning involved removing incomplete records, duplicate entries, or implausible values that included RT > 720 min/d, MY > 70 kg/d, WI > 150 L/d, based on biological plausibility. The final dataset was validated by cross-checking a random 5% sample against raw records to ensure accuracy. The THI during the study period was 68.5 ± 8.2 (range: 45.2–82.7), reflecting typical environmental conditions in central Texas. No significant data loss occurred because of sensor malfunctions or incomplete records after data cleaning.

Statistical analyses were performed using R (version 4.3.2, R Core Team, 2023) with the packages *lme4* (Bates et al., 2015) and emmeans (Lenth, 2025) for mixed-effects modeling and post hoc comparisons, respectively. The effects of PME on RT, MY, and WI were analyzed using linear mixed-effects models with repeated measures. Each outcome variable (RT, MY, WI) was modeled separately, with the following fixed effects: day relative to pen move (d −1, 0, 1, 2, 3, 4, 5), parity (primiparous or multiparous), stage of lactation (early, mid, late), THI (continuous variable), and move group size number (single, small, and large). To work with simpler models and account for model convergence issues, we developed 2 sets of models for each outcome variable (RT, MY, WI). The first set of models included parity, stage of lactation, and day relative to the PME, as well as their 2-way and significant 3-way interaction effects of parity × stage of lactation × day relative to the PME, while controlling for daily THI and random animal effects in the model. In the second set of models, we evaluated whether parity-specific effects on RT, MY, WI varied according to the number of cows moved together by including 2 way interactions as well as a significant 3-way interaction among parity × move group size (single, small, or large) × day relative to the PME, controlling for lactation stage, THI, and random animal effects. Three-way interaction terms were specified a priori because the primary objective of the study was to determine whether parity-specific responses to PME varied across days following the move and whether these temporal patterns differed by lactation stage or by move group size. Lower-order main effects and 2-way interactions were implicitly included within the 3-way interaction structure. Although both models included 2-way interactions of respective variables, we focus only on the significant 3-way interaction for the sake of brevity in this short communication. The covariance structure was selected based on the lowest Akaike information criterion and likelihood ratio test. Model assumptions were verified by examining residual plots for normality and homoscedasticity. Outliers were identified using a threshold of ±3 SD from the mean and they were removed if they represented data entry errors (n = 4). Post hoc pairwise comparisons were conducted using the Tukey method to evaluate differences with significance set at *P* < 0.05. Results are presented as estimated marginal means with SE.

A significant 3-way interaction among parity, lactation stage, and day relative to the PME was observed for RT (*F*_18,181988_ = 12.153; *P* < 0.01; Figure 1A). Across all lactation stages, multiparous cows exhibited a significant reduction in RT during the post-PME period (d 1–5) compared with their d −1 level. In early lactation, multiparous cows showed significant decreases in RT on d 2–4, corresponding to reductions of ∼3% to 4% relative to d −1 (*P* < 0.001). A similar, though less pronounced, response was observed in early-lactation primiparous cows, with RT decreasing by ∼3.6% on d 2 post-PME compared with d −1. The magnitude of RT reduction was greatest in dry multiparous cows, which experienced a progressive decline following the PME, reaching a maximum reduction of ∼9% by d 5 compared with their d −1 levels.

**Figure 1:**
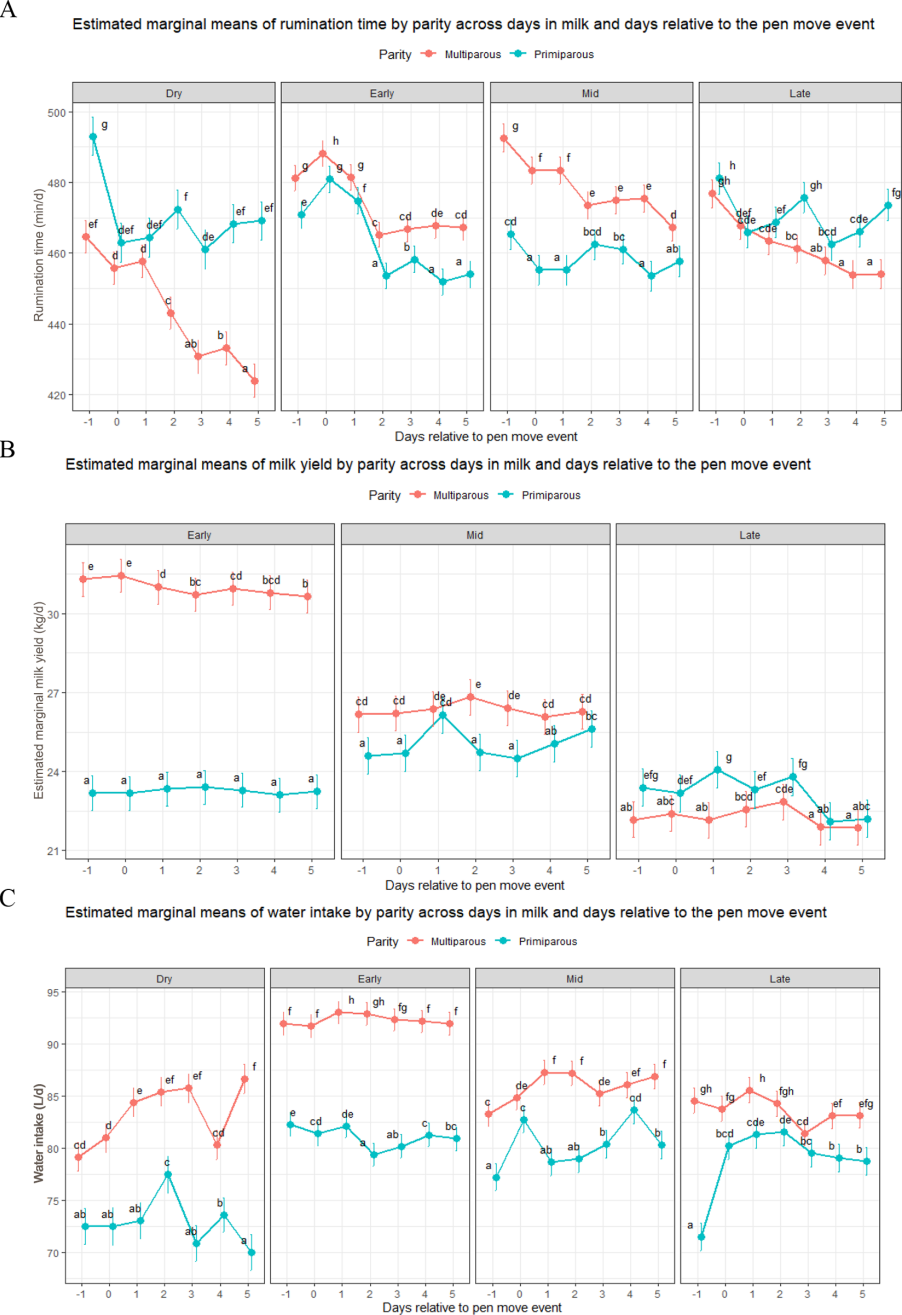
Estimated marginal means of (A) MY, (B) RT, and (C) WI for primiparous (red line) and multiparous (blue line) cows from day −1 to d 5 relative to PME. Error bars represent SE. Different lowercase letters indicate significant differences (*P* < 0.05).

A significant 3-way interaction between parity, lactation stage, and day relative to PME was detected for MY (*F*_18,185796_ = 20.626; *P* < 0.001; Figure 1B). In late lactation, primiparous cows exhibited significant reductions in MY on d 4 and 5 post-PME, corresponding to decreases of ∼1.9 and 2.0 kg/d (7.9% and 8.3%), respectively, compared with their d −1 levels. In contrast, no significant post-PME change in MY was observed for multiparous cows in late lactation. In early lactation, multiparous cows showed a consistent and significant decline in MY across all post-PME days (d 1–5), with reductions ranging from ∼0.6 kg/d (1.9%) in the early post-PME period to 1.9 kg/d (6.3%) by d 5. No significant post-PME decrease in MY was observed in early-lactation primiparous cows.

Similarly, a significant 3-way interaction between parity, lactation stage, and day relative to PME was found for WI (*F*_18,226836_ = 39.19, *P* < 0.01; Figure 1C). In early-lactation primiparous cows, WI decreased significantly during the PME period, with reductions observed on d 2–5 relative to d −1 (*P* < 0.01). The magnitude of the decrease was greatest on d 2 (∼4.7 L/d; 5.6%) and progressively attenuated over subsequent days, with reductions of ∼3% to 4% on d 3 and 3% on d 4 and 5. In contrast, multiparous cows exhibited a transient increase in WI on d 1 and 2 post-PME compared with their respective d −1 levels in the dry period, early lactation, and mid lactation.

A significant interaction effect on RT was also observed for the PME days, parity, and movement group size (*F*_12,182242_ = 35.53; *P* < 0.01; Figure 2A). Overall, a decrease in RT was observed after PME days in primiparous and multiparous cows for single cows, small group, and large-group PME. However, the magnitude of RT decrease for primiparous cows was greater for the cows moving in small and large groups compared with single-cow moves. Results (Figure 2A) indicate that primiparous cows moved in larger groups were more adversely affected by PME. Specifically, a significant decrease in RT from d −1 to d 1 (*P* < 0.01) was observed in primiparous cows moved individually or in small groups, with the decline persisting through d 4 in large-group moves. Multiparous cows also showed a significant reduction in RT across all single cows, small-group, and large-group PME.

**Figure 2:**
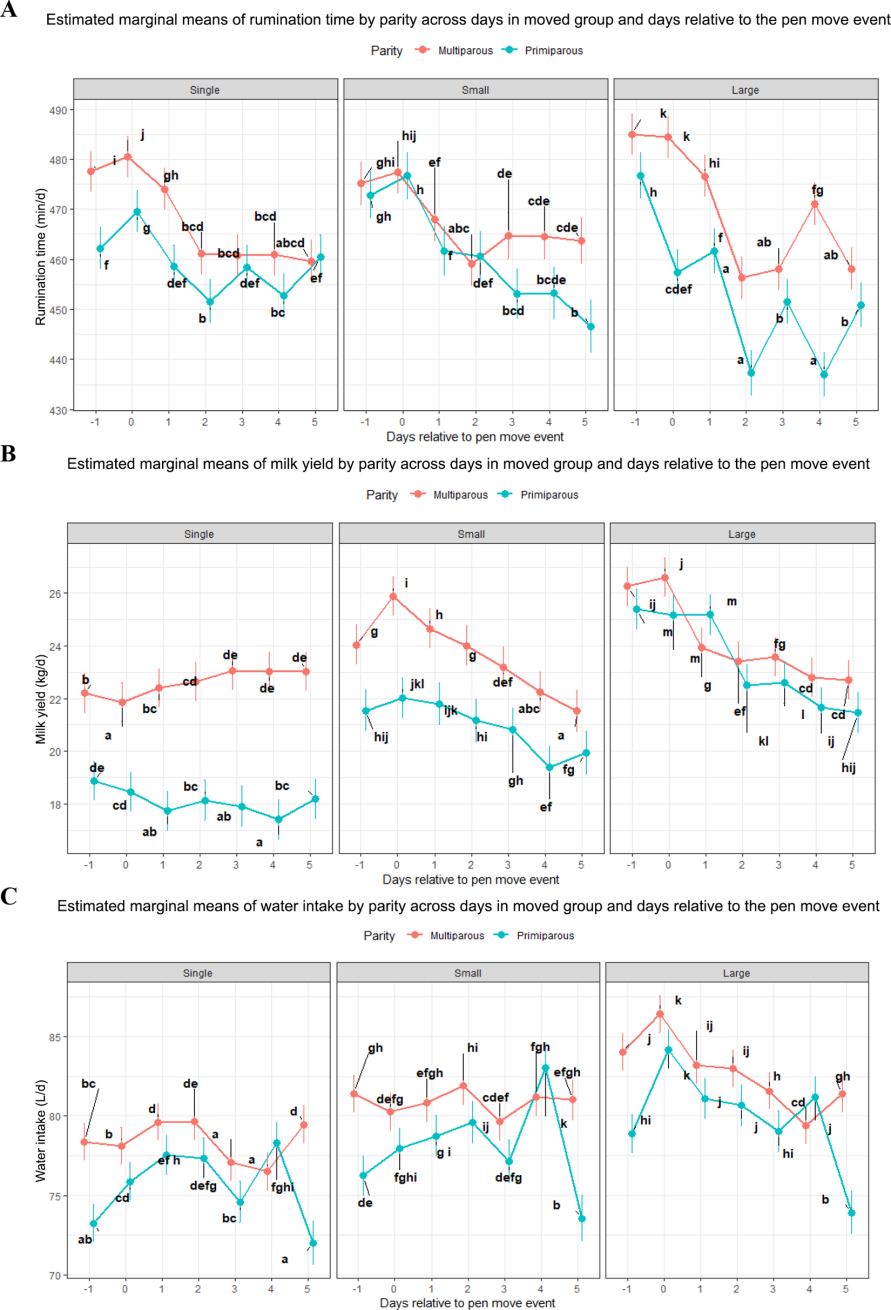
Estimated marginal means of (A) MY, (B) RT, and (C) WI across days relative to the PME by parity and moving group size. Error bars represent SE. Different lowercase letters indicate significant differences (*P* < 0.05).

A significant interaction effect on MY was observed (*F*_12,185933_ = 32.72; *P* < 0.01; Figure 2B) for days relative to PME, parity, and group size. For single-group PME, no significant change in MY was observed across days relative to the PME (d −1 to 5) in primiparous cows. In contrast, multiparous cows exhibited a transient reduction in MY on the day of the PME (d 0), followed by a recovery to premove production levels by d 2. A decrease in MY was observed across both small and large group size PME in both parity groups (Figure 2B). In both small- and large-group moves, the decrease in MY for multiparous cows was more pronounced compared with primiparous cows, representing a difference in the effect of PME by group size and parity.

A significant interaction between group size, parity, and day relative to PME was observed for WI (*F*_12,186102_ = 25.03; *P* < 0.01; Figure 2C). For cows moved in large groups, WI was increased on d 0 relative to PME and significantly reduced from dv0 levels on all days post-PME for both primiparous and multiparous cows (*P* < 0.05). For single-cow moves and small-group moves, we observed increased WI up to d 2 before a subsequent decrease in both primiparous and multiparous cows.

The results demonstrate distinct effects of PME on RT, MY, and WI for primiparous and multiparous cows in different stages of lactation. These findings highlight the complex interplay between social stressors, parity, and physiological outcomes, offering insights into dairy cow welfare and management practices. The observed reductions in RT post-PME, particularly pronounced in multiparous cows in all stage of lactations and in early lactation in primiparous cows, align with previous research on social stress in dairy cows. Scheurwater et al. (2021) found a 1 min/h decrease in RT following cow move events, with effects persisting during stressful conditions like estrus. The pronounced and sustained reduction in RT observed among both primiparous and multiparous cows in our study was explained by the findings of Marumo et al. (2024), who reported that younger cows experience behavioral disruptions following regrouping, likely because of their lower social rank and limited prior experience with group changes. Schirmann et al. (2011) reported a reduction in DMI on the day of regrouping, which is closely linked to rumination. Mazer et al. (2020) observed a higher fecal cortisol level in primiparous cows postregrouping, attributed to increased agonistic interactions at pens. Similarly, Marumo et al. (2024) found that regrouping prepartum cows led to a 15 to 20 min/d decrease in rumination, particularly when moved into pens with established hierarchies. These reductions likely reflect stress-induced suppression of rumen motility, as noted by Herskin et al. (2007), who linked social stressors to altered gastrointestinal function. Proudfoot et al. (2012) linked social dynamics to disease, connecting PME cows between groups to increased disease risk. Our results demonstrate that RT disruptions persist beyond the immediate move day, in both primiparous and multiparous cows in single, small, and large move groups, indicating stress and vulnerability to disease because a decrease in RT is generally associated with suboptimal health (Paudyal et al., 2018).

Primiparous and multiparous cows also responded differently following PME on MY. Primiparous cows in late lactation had a significant decrease (4%) in MY on the fourth day after PME; however, in multiparous cows, there was a consistent reduction in early lactation from the first day to the fifth day (3%). A study by Marumo et al. (2024) reported that there was a reduction of 12.2% of MY in primiparous cows on the day after moving the cow, in contrast with multiparous cows being less affected by the reduction in MY. However, similar to our finding, Zwald and Shaver (2012) reported that mid-lactation cows experience minimal negative effects of PME on MY.

Golher et al. (2021) indicated that WI is influenced by several factors, including breed, parity, and milk production, as observed in this study. Water intake responses to PME strongly varied by parity, with early lactation primiparous cows showing reductions of 4.2 L/d on the second day after the move and reductions of 3.36 L/d on d 3, 2.49 L/d on d 4, and on d 5, a 2.61 L/d, possibly suggesting an issue with access to the drinking water associated with social hierarchy and pen dynamics changes after the PME (Woodford et al., 1984). Primiparous cows also demonstrated a greater drop in WI in single, small, and large pen moves relative to d −1 levels compared with multiparous cows, especially on d 5 post-PME, representing an effect across all pen move sizes. These parity-effect patterns are supported by findings from McDonald et al. (2020), who also observed a drop in WI, pronounced in primiparous cows, similar to our observations on d 5 post-PME.

The effect of PME group size in this study indicated a greater impact with large-group moves. Foris et al. (2021) suggested that cow familiarity influences cow social network after pen moves and regrouping, indicating the move group size has an effect on the establishment and maintenance of social hierarchy in cattle. In contrast to our findings, Mazer et al. (2020) indicated that individually moved cows in smaller groups experience higher stress conditions as indicated by high fecal cortisol levels. This may have been the result of the smaller pen sizes and move groups in these studies compared with our study, where a single-cow move, when moved to a group of 300 animals, may go unnoticed compared with large groups that move with >7 cows, demonstrating noticeable changes. Lobeck-Luchterhand et al. (2014) studied cow-regrouping strategy in prepartum cows and identified that cows regrouped less frequently were associated with longer feeding times. The contradictions in findings may be attributed to the use of crossbred cows in the present study, whereas previous research primarily involved Holstein cows. The genetic and physiological differences between these breeds could contribute to the observed discrepancies. However, this hypothesis warrants further investigation.

These findings have practical implications for dairy farm management. The significant RT and MY reductions suggest that minimizing PME or optimizing their timing (e.g., avoiding early lactation or high-THI periods) could enhance cow welfare and productivity. Strategies such as gradual introductions (Talebi et al., 2014) or maintaining stable social groups (Grant and Albright, 1995) may mitigate stress. Cabrera and Kalantari (2016) conducted an economic evaluation related to pen moves and multiple TMR management practices. Results indicate that multiple TMR groups economically outperform no pen moves using single TMR, even after considering plausible potential milk losses when grouping (Cabrera and Kalantari, 2016). When making these decisions, the positive effects of regrouping, including improved herd health and environmental stewardship, should be compared against additional costs of management, labor, facilities, and equipment required for regrouping and pen moves, which are specific to the farm. Contreras-Govea et al. (2015) reported that milk production declines following cow move and regrouping as a primary factor for farmers to have a limited number of feeding groups, alongside restrictions from farm facilities and available labor.

It is important to recognize the potential effect of overcrowding on the variables monitored in this study. Barrientos-Blanco et al. (2022) investigated the effects of pen overcrowding and reported a 3% to 6% reduction in DMI when stocking density exceeded recommended levels by 30%, that is, 1.3 cows per head gate. Although the exact stocking density of the pens during the study was unknown, the goal of the farmer was to maintain a 100% stocking rate. Even though data reports from DairyComp followed established farm protocols emphasizing accuracy, such as double-checking entries and ensuring consistency in dates and critical information, manual data entry by farm personnel inherently carries a risk of minor inaccuracies. These potential errors, although minimized through standard procedures, cannot be completely eliminated and should be considered when interpreting the study findings. This study benefits from a substantial cow population and comprehensive data collection, but the conclusions from this study should be interpreted within the context of this farm. The research was conducted on a single organic dairy farm using crossbred cows, and although the data from the grazing period was excluded to reduce potential confounding effects, measurements may be different in conventional operations with differing management practices. We also did not evaluate long-term effects beyond 5 d postmove, which could reveal recovery patterns or chronic stress effects.

In summary, this study demonstrates that PME significantly influence RT, MY, and WI in dairy cows, with notable differences based on parity and group size. These results highlight the importance of maintaining stable social groups and the potential for using monitoring technologies to evaluate these effects. Future research should explore long-term effects and validate findings across diverse farm systems to further inform management strategies aimed at better dairy cow welfare and productivity.
